# Supplementary material for: Consistency in the Assessment of Dried Blood Spot Specimen Size and Quality in U.K. Newborn Screening Laboratories
Source: Int J Neonatal Screen. 2024 Sep 5;10(3):60. doi: 10.3390/ijns10030060 (PMC11417764; doi:10.3390/ijns10030060)

Supplementary File 1.

Blood Spot Quality Guidelines for Screening Laboratories 2015

The problem: The avoidable repeat rate for blood spot cards varies across the country. One cause is variability in the rejection criteria used by newborn screening laboratories. These guidelines are based on blood spot quality research and audit, and describe the agreed rejection criteria to be used by English newborn screening laboratories. They are intended to increase the quality of blood spot samples and, in turn, the efficiency of the screening pathway.

Background: The need to standardise blood spot quality acceptance and rejection criteria was acknowledged at the UK Newborn Screening Laboratory Network (UKNSLN) meeting in June 2014 following work that had been carried out by some members of the group. It was agreed by the laboratory directors that a national approach would be needed to address the high avoidable repeat rate in some areas. It was also recognised that a low avoidable repeat rate did not necessarily mean better quality samples as different laboratories accepted varying quality of samples. These guidelines have been produced with the aim of standardising the acceptance and rejection criteria, resulting in accurate avoidable repeat rates that are comparable between laboratories, and will enable services to lower the avoidable repeat rate.

The UKNSLN agreed in June 2014 that each single spot must be filled with a minimum spot diameter of blood of 7mm. The acceptable number of spots which meet this criteria is at the discretion of the screening laboratory. Each 7mm diameter spot will provide 2 discs for testing. Blood spots less than 7mm diameter are not suitable for testing due to the low volume of blood in these samples (approximately 10µL or less). The smaller the volume the greater the negative bias on results for all newborn screening analytes (R. George & S. Moat 2014). Measuring each spot dimension is not practical when processing the cards as not all blood spots are circular therefore an acceptable spot is where 2 discs can be punched from it (each disc is 3.2mm).

The date for implementation of the guidelines by laboratories is 1st April 2015. The blood spot programme is preparing education and training materials for maternity and health visiting services and a communication strategy to support implementation.
Midwives should send a card with four good quality spots and completed demographic information. The screening laboratory will use the following criteria to accept/ reject samples. Whilst this guidance is extensive it is by no means comprehensive. The laboratory has the right to reject any sample where it considers the ability to produce the correct screening result is being compromised by the condition of the blood spot.

References:

L. Hamilton, '*Bloodspot Sample Quality Project'*, Central Manchester University Hospital, April 2013

M. Downing, *'Quality and Improvement'*, Sheffield Childrens NHS Trust, February 2014

R. George & S. Moat, *'Bloodspot Quality Project Report'*, Wales Newborn Screening Laboratory, September 2014

S.K. Hall, *'Solving the non-equivalence between laboratories' spot acceptance criteria. Strategy for improving quality of newborn bloodspots. Discussion document for QA teams',* June 2014

S.K. Hall, '*Blood Spot Quality'*, Birmingham Children's Hospital, November 2013 UKNSLN, '*First Bloodspot Quality and Luminex Cardscan Meeting'*, October 2013

Unacceptable Samples

Unsuitable sample – incorrect blood application	Incorrect blood application technique – for example spotting on both sides or several small spots to fill one whole circle will comprise of areas where the blood has not penetrated the filter paper.<br>As the sample is not homogeneous, results are dependent on the area of the sample punched. Such samples have been found to produce lower analyte results compared to single spot samples (R. George & S. Moat 2014, L. Hamilton 2013). Therefore, the higher risk of false negative results associated with these samples means they are not suitable for testing.

	Status code 0304
Unsuitable samples – compressed or damaged spots	Compressed samples are not suitable for testing due to the significantly higher risk of false negative results associated with them. For some analytes the results can be up to 45% lower (R. George & S. Moat 2014, L. Hamilton 2013). This can be seen by evidence of incomplete drying, stained glassine, scratched/abraded/ridged spots, evidence of liquid/water contamination or discoloured spots.<br>*Status code 0305.*
Other unacceptable samples	Too young for reliable screening - *Status code 0301*<br>Too soon after transfusion (<72 hours)- *Status code 0302*<br>Contaminated (e.g. Samples suspected of dilution of infusate; adult blood) – *Status code 0305*<br>Unsuitable sample: day 0 and day 5 on same card – *Status code 0306*<br>Contaminated (e.g. faeces) -*Status code 0307*<br>Missing/not accurately recorded demographic details (NHS number*, date of sample, date of birth) - *Status codes 0308, 0309, or 0310*<br>Expired blood spot card used - *Status code 0311*<br>Blood spot cards more than 14 days in transit - *Status code 0312*<br>Unsuitable sample: Damaged in transit (includes water/liquid damage through outer postal envelope) – *Status code 0313*<br>Too old for screening (>1 year) – *Status code 0902*<br><br>* day 0 samples may not yet have an NHS number

Acceptable Samples

Each spot	Each spot is large enough to punch two discs[1] (each disc is 3.2mm diameter) from each spot. Punching from small blood spots can result in a reduced concentration of the metabolites to be tested.<br><br>If screening cannot be completed because the spot is not large enough for punching two discs, the card is to be classed as insufficient and rejected. The laboratory will request a repeat sample. In this case *Status code 0303* is used
Multi-layered spots	Multi-layered samples can be accepted provided they are not spotted on both sides of the card. The greater volume of blood in these samples will result in a positive bias. Though this would raise the number of false positives slightly, the risk of missing a positive case is reduced (R. George & S. Moat 2014, L. Hamilton 2013).

Supplementary File 2.

PROCEDURE FOR ASSESSING BLOODSPOT SPECIMEN ACCEPTABILITY FOR NEWBORN SCREENING

1.0 Introduction

1.1 Scope and Purpose

The purpose of this standard operating procedure (SOP) is to ensure that there is a standardised approach for assessing dried blood spot size and quality, thereby ensuring satisfactory newborn screening analyte results. Adherence to this SOP should ensure that blood spot specimens that are unsuitable for analysis can be appropriately identified and a repeat specimen promptly requested.

All specimens received into the laboratory should be analysed, even if they are deemed unsuitable. Any screen positive result(s) should be acted upon as per relevant screen positive protocol(s) even if the result was obtained from an insufficient sample and / or where the result(s) cannot be confirmed in duplicate due to poor quality or insufficient sample for analysis.

1.2 Definitions

Good quality specimen: A specimen that contains sufficient blood, which has been applied correctly to the filter paper, to allow testing for ALL conditions (including confirmatory and second tier tests).

Avoidable repeat: Specimens that are unacceptable for testing due to unacceptable blood spot size, quality, insufficient, delay in transit or incorrect demographics on the card.

1.3 Responsibility

All UK Newborn Screening Laboratories should follow the guidance in this SOP.

1.4 Related Documents

- Blood spot quality assessment competency training log.
- Blood spot quality assessment observation / competency form.
- Blood spot quality competency assessment (set of DBS Images).

1.5 References

Guidelines for Newborn Blood Spot Sampling (Public Health England). https://www.gov.uk/government/publications/newborn-blood-spot-screening-sampling-guidelines

Guidance - Newborn blood spot screening: laboratory guide for IMDs (Public Health England). https://www.gov.uk/government/publications/newborn-blood-spot-screening-laboratory-guide-for-imds

CLSI. Dried blood Spot Specimen Collection for Newborn Screening. 7th edition. CLSI standard NBS01. Clinical and Laboratory Standards Institute; 2021.

George, R.S. and Moat, S.J. Effect of Dried Blood Spot Quality on Newborn Screening Analyte Concentrations and Recommendations for Minimum Acceptance Criteria for Sample Analysis. Clinical Chemistry 2015; 62(3): 466–475.

Moat SJ, Dibden C, Tetlow L, Griffith C, Chilcott, Hamilton L, Wu THY, MacKenzie F, Hall SK. Effect of blood volume on analytical bias in dried blood spots prepared for newborn screening external quality assurance. Bioanalysis 2020, 12(2): 99-109.

Winter T, Lange A, Hannemann A, Nauck M, Muller C. Contamination of dried blood spots – an underestimated risk in newborn screening. Clin Chem Lab Med 2018; 56(2):278-284.

2.0 Procedure

2.1 Specimen Requirements

The table below outlines the criteria for blood spot specimen collection and handling:

**Sample Type**	Heel prick blood collected directly onto a bloodspot card. The card must be within the expiration date.
**Volume**	4 circles filled and evenly saturated with a single drop of blood. Blood must completely soak through the filter paper.
**Other Criteria**	Blood spots must be allowed to dry sufficiently before being placed in the glassine envelope. Samples must NOT be placed in plastic bags.
**Transport to the Laboratory**	Ambient temperature, 1st Class Post / courier service.
**Sample storage:**	As per local protocols.
**Storage Duration (Post-analytical)**	Screening cards: Follow UK guidance.
**Other Comments**	Blood containing citrate/EDTA cannot be used due to chelation of the europium label used in the TSH & IRT assays. Lithium heparin can inhibit the DNA assays.

The process of DBS specimen collection involves the application of a non-volumetric amount of blood onto the filter paper marked with printed guide circles. A single hanging drop of blood

of adequate size to fill the printed circle (10mm) should be applied to the filter paper as over or under filling the pre-printed circle affects the volume of blood in the sub-punch that is used for analysis. Blood should be applied to the front of the card only and the blood should penetrate through to the back of the paper. Small samples (<7mm diameter), multi-layering, multi-spotting or compression of the specimens can adversely affect the concentration of the analytes within the DBS leading to inaccurate results.

Education, training and ongoing competency assessment is of paramount importance to ensure that appropriately sized and good quality specimens are used for analysis, thereby ensuring accurate screening results.

2.2 Criteria for Acceptable and Unacceptable Blood spot Size and Quality

The quality of DBS specimens received into the laboratory for analysis should be assessed subjectively by visual inspection; ensuring that the printed circle (10mm diameter) is suitably filled with blood; that the blood is spread symmetrically and evenly on both sides of the filter paper. Laboratories should follow these guidelines to ensure a standardised approach for specimen acceptance/rejection in the UK.

Filter paper collection cards for capillary blood collection are Class II Medical Devices and should meet international criteria for performance. These collection devices should not be used after the expiry date printed on the device.

The expiry date of the card should be checked on receipt into the laboratory to ensure it is within the expiration date. If the specimen is collected before the expiry date but then arrives into the lab after this date (but within 14 days) this specimen should be accepted.

During transportation, the DBS specimens can be exposed to extreme environmental conditions (e.g. high temperatures and high humidity). The stability and extraction of the analytes can be affected in older specimens. **Those specimens that have been in transit >14 days should be rejected.**

Where specimens are to be rejected a second opinion should be sought to ensure consistency of sample rejection within the laboratory.

2.3 Acceptable Blood spot Quality

Below is an example of a good quality specimen – the specimen has been formed by a single hanging drop of blood, appropriately filling the pre-printed circles and with both sides of the paper evenly saturated.

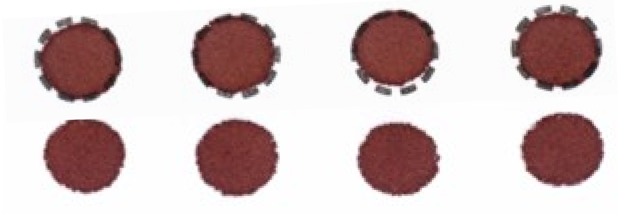

Front of card

Back of card

Acceptable blood spots must meet the following criteria:

Any individual blood spot used for ***reportable*** testing must meet the quality standards outlined in this document. If there is sufficient blood of acceptable quality to complete all testing safely from the specimen provided, then a repeat sample is not required (unless indicated for any other reason not related to spot quality).

If testing for all conditions, including any required re-tests and second tier testing indicated from the initial screen positive result, cannot be completed from bloodspots meeting the criteria outlined, then a repeat card must be requested.

If a lack of a sufficiently good quality specimen becomes apparent (i.e. failed second tier testing) after an interim report has been generated (depending on local IT processes) then it is not necessary to amend the report to reject the already reported condition(s). The remaining outstanding test(s) should be completed on the repeat specimen without the requirement to repeat any previously reported tests(s).

Blood spots must be of sufficient size to allow at least TWO sub-punches to be taken from each spot. In practice, circular spots >7mm in diameter (formed from a single hanging drop of blood) allow a minimum of 2 sub-punches to be taken. Those specimens where a minimum of 2 sub-punches cannot be taken from a single spot should be rejected as such samples produce significant negative biases for the analytes tested.

The following examples below demonstrate the minimum acceptance criteria:

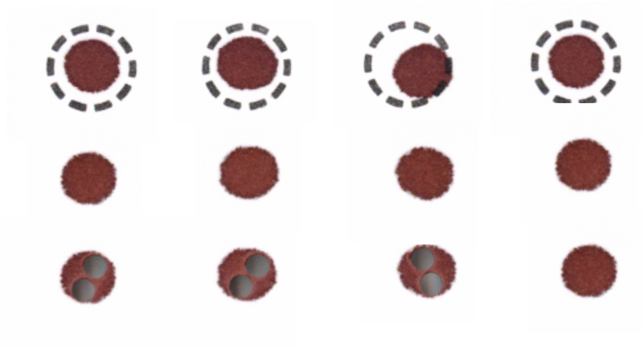

Able to take 2 sub-punches from a single spot

In the example below the blood spots are overlapping. It is recommended that the sub-punches are taken from the edge and NOT from the overlapping area.

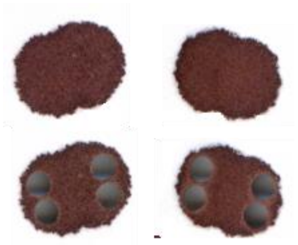

Spots touching - able to take 2 sub-punches from each spot at the edge, without taking punches from the overlapping area of the two spots.

In the example below; spot 1 is sufficiently large enough to enable 5 sub punches to be taken for the intial screening (IMDs, TSH, IRT, SCD & SCID screening). The remaining 3 blood spots are <7mm and are unsuitable.

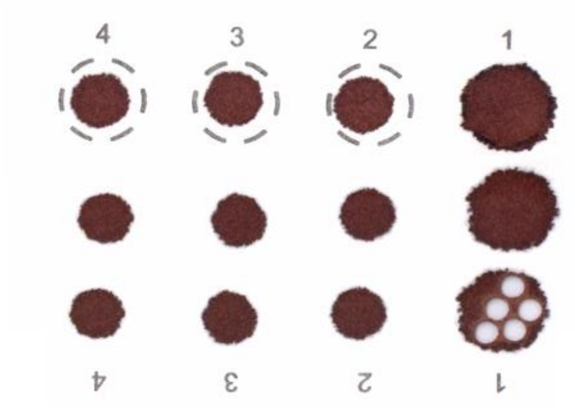

1 spot adequate (>7mm but <14mm and at least 5 sub-punches can be taken) – All other spots - unable to allow 2 sub-punches to be taken.

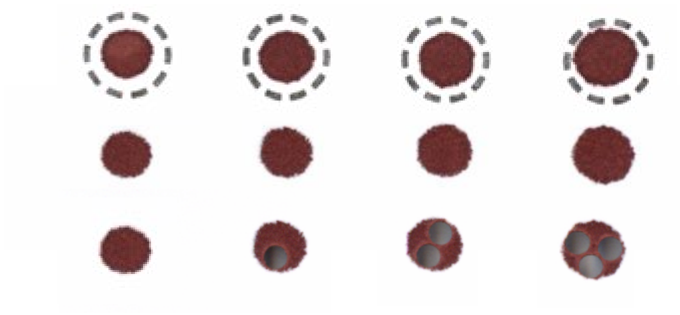

2 spots adequate (i.e. 3 sub-punches can be taken from 1 spot and 2 from another spot).

For non SCID screening laboratories (only 4 sub-punches are required) the following specimens would be deemed acceptable.

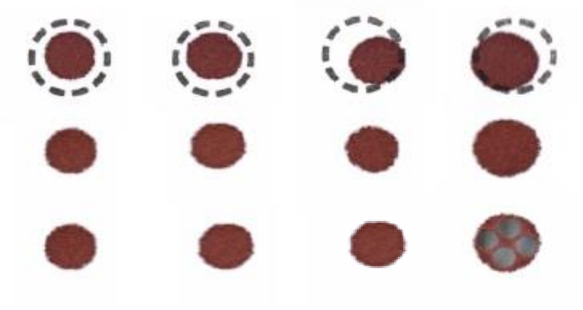

1 spot adequate (>7mm but <14mm and 4 sub-punches can be taken) – All other spots <7mm - unable to allow 2 sub-punches to be taken.

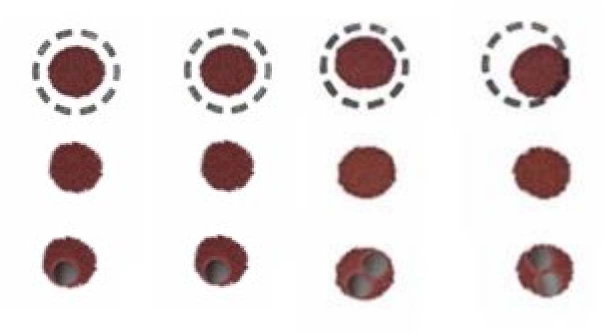

2 spots adequate (i.e. able to take 2 sub-punches from 2 spots) – All other spots unable to take 2 sub-punches from a single spot – Specimen can be accepted.

In the examples below the blood has uniformly permeated on both sides of the filter paper, in addtion there is no evidence of multi-spotting (see examples in section 2.4.3 on p 8) and such samples would be deemed acceptable where 2 or more subpunches can be taken from each spot.

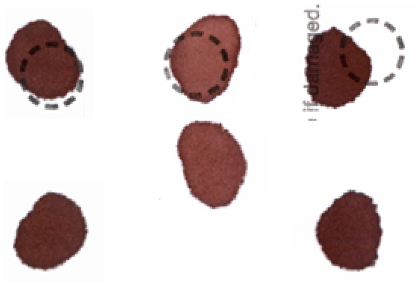

NB – day 1 cards for SCD screening, day 28 TSH testing and family history IMD screening require only 1 good quality spot.

Unacceptable bloodspot quality

2.4.1 Insufficient samples – Unable to take 2 sub-punches from a single spot

Blood spots too small i.e. <7mm. Such specimens produce falsely low analyte concentrations. The images below show examples of specimens that should be rejected:

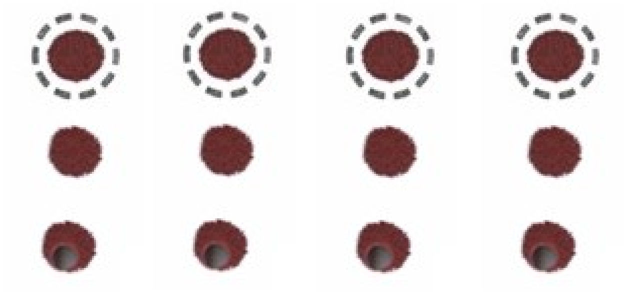

Unable to take 2 sub-punches from a single spot

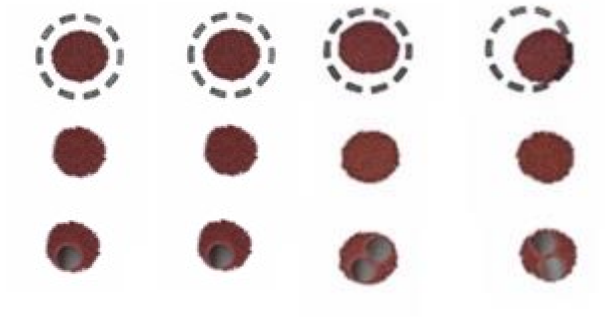

SCID screening lab

2 spots adequate (i.e. able to take 2 sub-punches from 2 spots) – All other spots unable to take 2 sub-punches from a single spot – Specimen must be rejected.

2.4.2 Insufficient samples – blood not soaked through to the back of the filter paper.

Blood sample has not saturated and uniformly penetrated the filter paper. Such specimens are formed when the sample taker has not achieved the formation of a hanging drop of blood and places the heel against the filter paper, preventing the blood permeating the filter paper. Such specimens produce falsely low analyte concentrations.

The images below provide examples of insufficient specimens and should be rejected.

Front of card

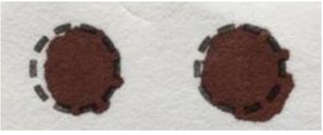

Back of card

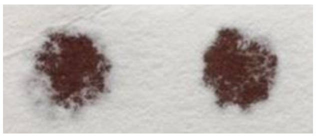

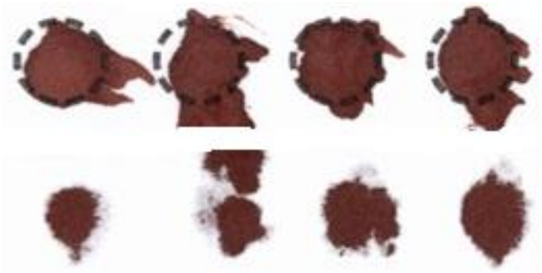

All spots unsuitable – Heel has been placed against filter paper or baby may have kicked when applying a non - hanging drop of blood.

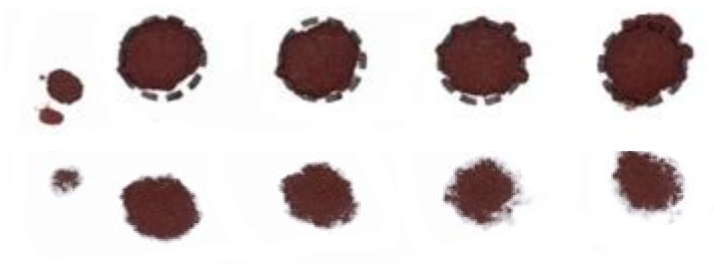

All spots unsuitable – The blood has not evenly saturated the filter paper. Hanging drop of blood was not formed and samples have been smeared or ‘painted’ into the circle by applying heel against the paper. Also evidence of multi-spotting.

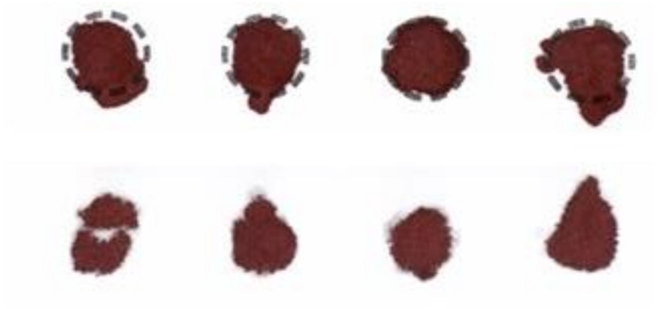

NB – Specimens with a low haematocrit (Hct) are more symmetrical with smooth edges, whereas those with a higher Hct are smaller, darker and have an uneven edge - See Appendix for examples.

2.4.3 Unsuitable specimen (blood quality) – incorrect blood application technique; includes multi-spotted and merged / overlapping spot, spotted both sides & multi-layered (excess blood application) and clotted specimen.

Multi-spotted specimens

Multi-spotted and insufficient (samples do not have a circular edge and the edges of each individual spot can often be seen within the spot).

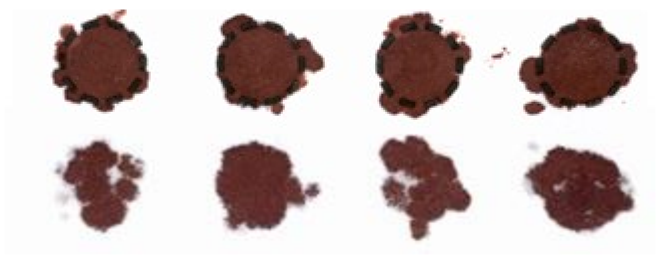

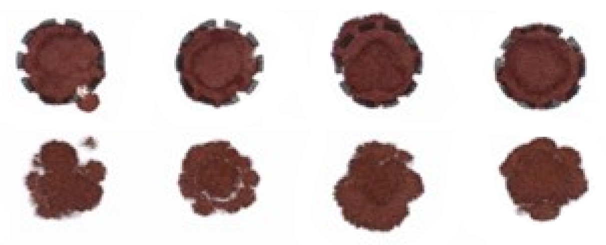

All spots are unsuitable - multi-spotted (multiple small spots used to create one large spot)

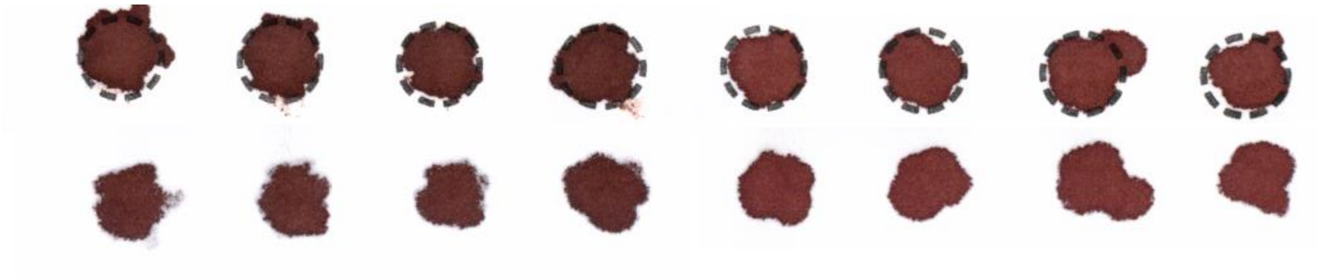

Blood application to both sides of filter paper

Blood applied to the front AND back of the filter paper where the two applications do not overlap (See image below). Such specimens produce heterogeneous results and should be rejected.

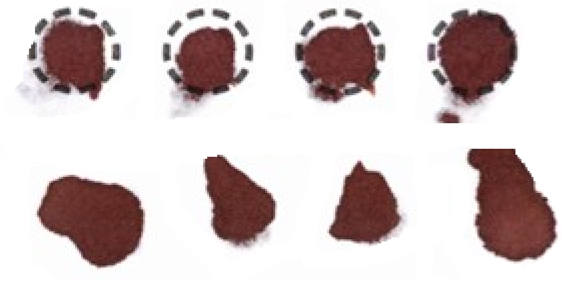

Front of card

Back of card

It can be difficult to determine if a sample has been applied to both sides of the card. The image below shows a specimen prepared in the lab by applying 30µL volumes of blood to both sides of the filter paper. The blood has uniformly permeated the filter paper on both sides and such a sample would be deemed acceptable.

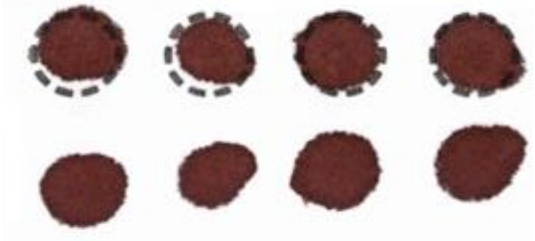

Excess blood application (? Multi-layered specimens)

Such specimens are usually the result of blood being applied from a line, syringe or the use of an inappropriate lancet.

Overfilling the pre-printed circle with blood results in significant positive biases. Such specimens can result in false positive results especially for those disorders where affected specimens may be near to cut-off values (e.g. TSH, Leucine & C5DC).

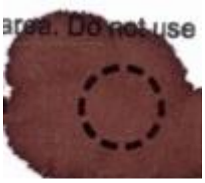

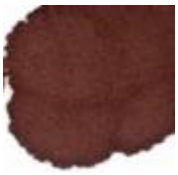
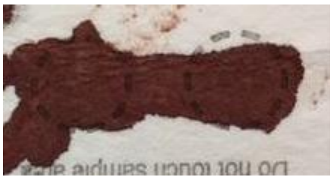

In order to provide consistency in the rejection of specimens where excess blood has been applied. It is recommended that those specimens that contain blood spots >14 mm should be rejected. A spot diameter >14 mm is too large to have come from a single drop of blood and should be rejected (see image below).

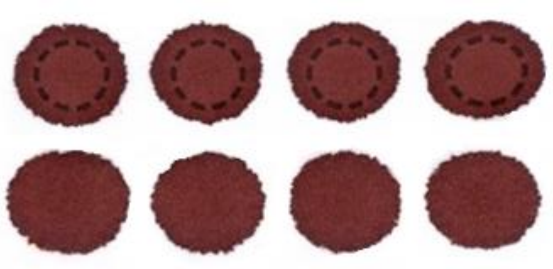

Clotted Specimen

Clotting may be observed when multiple small drops of blood are applied over too long a period of time or when using a capillary tube too slowly and such specimens should be rejected.

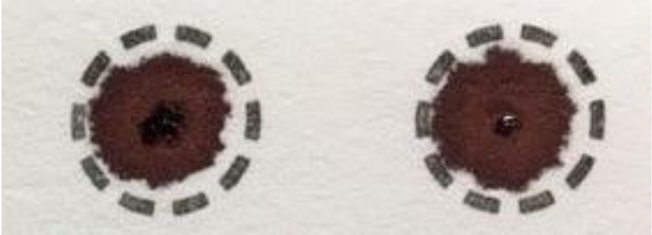

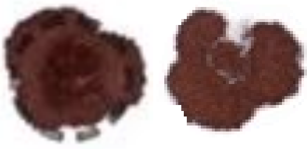

2.4.4 Unsuitable specimen (blood quality) – specimen compressed or damaged; includes compressed, evidence of incomplete drying, blood stained glassine envelope, scratched / abraded / ridged, liquid / water damage / contamination, includes discoloured spots

Compressed specimen

Evidence that pressure has been applied to the bloodspot - compressed spots have a pale centre with a darker ring around the edge. If there is evidence that a specimen has been compressed (see image below) and/or there is blood on the glassine envelope then these specimens MUST be rejected as there is a significant risk of missing a disorder.

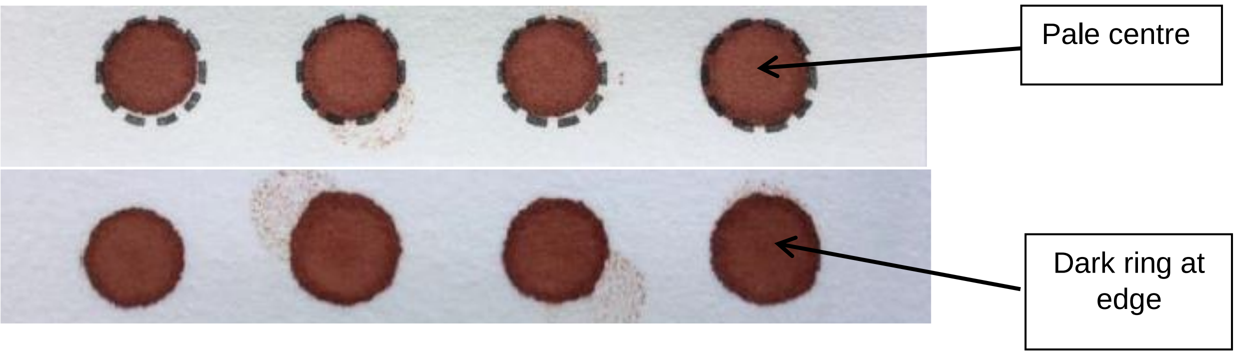

Incomplete drying of specimen

Specimen placed in glassine envelope when wet (mostly due inadequate drying or layered sample applied causing crinkling/ ridging of the filter paper). Any specimen received where blood is observed on the glassine envelope should be rejected i.e. if there is blood on the glassine envelope that corresponds to ALL 4 spots. If there are at least two spots on the card that have not stained the glassine envelope and are of good quality and size – then the sub-punches should be taken from these spots.

Front of card

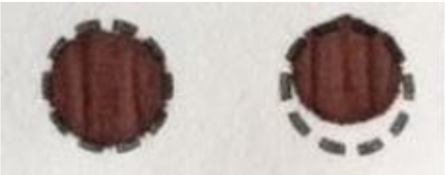

Glassine envelope

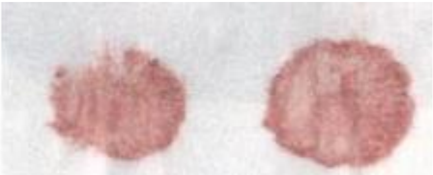

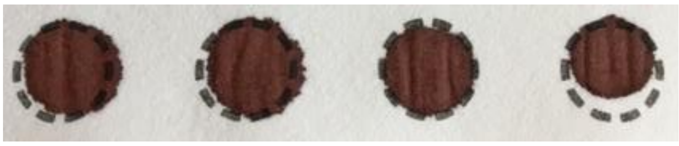

All spots are unacceptable – no evidence of blood on glassine envelope.

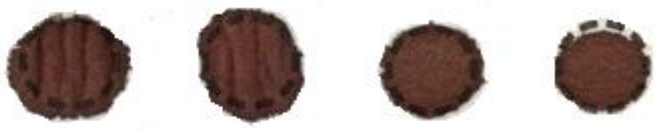

Spots 1 & 2 are unacceptable – Spots 3 & 4 are acceptable and specimen can be accepted for analysis.

Specimen dried in contact with a solid surface

Specimen has been placed against a solid surface after collection and before being place into the glassine envelope. NB – There may not be evidence of blood on the glassine envelope. Spots below exhibit patches where the blood has dried against a solid surface and such specimens should be rejected.

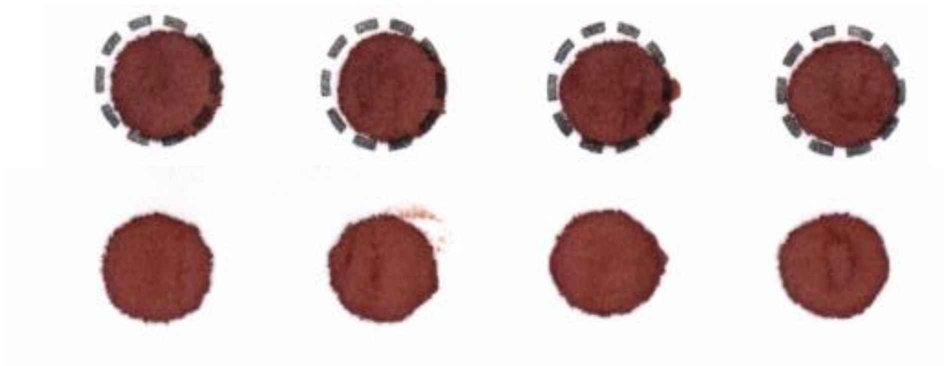

Blood spot specimen scratched or abraded (possible use of capillary tube). Any evidence that the specimen is scratched or abraded must be rejected (such samples are usually seen from neonatal units).

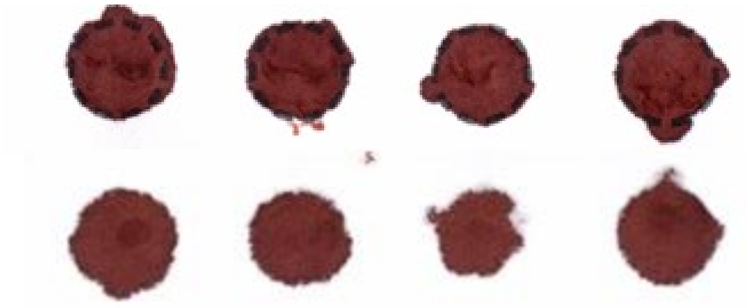

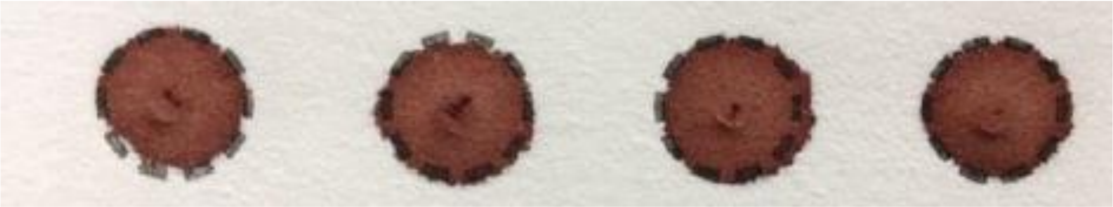

Contaminated specimen

Any evidence that the specimen was contaminated before or after collection, such specimens should be rejected and a repeat specimen collected. The following examples demonstrate contamination:

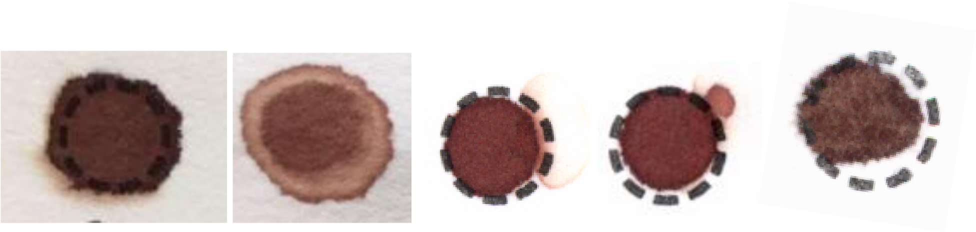

NB – Placing the specimen under a UV lamp may help to identify any contamination e.g. vaseline that is often used on neonatal units to collect capillary blood from a heel prick.

Blood specimen exhibits serum rings

This can occur if the filter paper has come into contact with alcohol, water, hand/body lotion or the puncture site has been excessively squeezed and such specimens should be rejected.

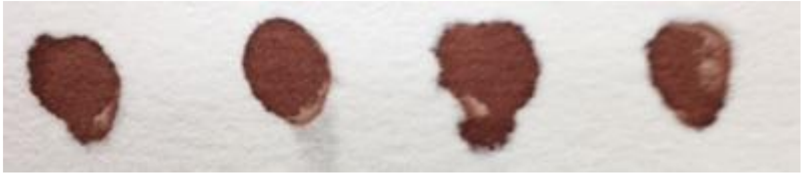

2.4.5 Unsuitable sample for Cystic Fibrosis screening: possible faecal contamination

Specimens that have evidence of faecal contamination must be rejected as this can lead to falsely elevated IRT concentrations as well as increases in C5-DC and C8. Images below show examples of faecal contamination:

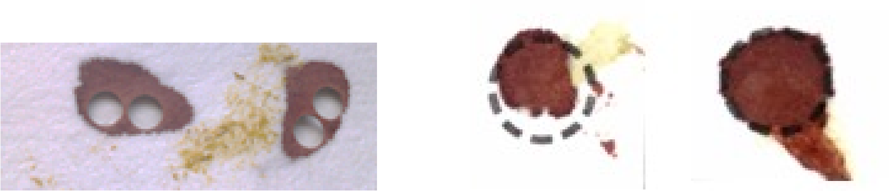

Those specimens that have no evidence of faecal contamination but have discrepant IRT values then a repeat specimen should be collected. NB - it is not necessary to repeat the analysis of the other screening analytes.

NB – Contamination (e.g. faecal, urine or infant feeds) may not be visible - discrepant analyte results may be indicative of contamination and a repeat specimen should be requested. See Table in Appendix outlining the analytes affected by various sources of contamination.

2.4.6 Unsuitable specimen: damaged in transit

Any specimen with evidence of exposure to moisture or water/liquid damage to any part of the card, filter paper or postal envelope should be rejected as specimens subjected to moisture / high humidity result in the rapid degradation of analytes within 24 hours.

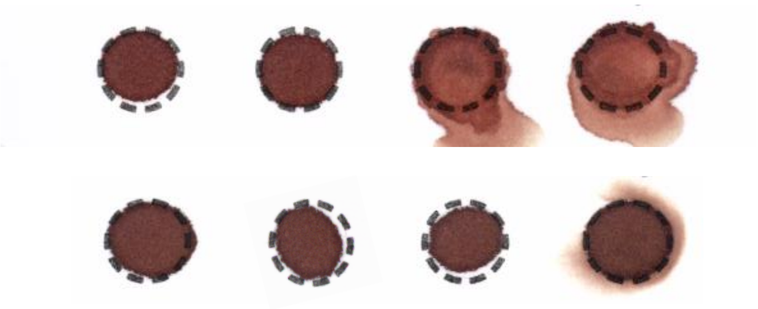

Specimens should be rejected as evidence of moisture exposure / contamination

3.0 Requesting Repeat Specimens

If the laboratory receives a specimen that is deemed to be unacceptable due to blood spot size, quality or contaminated, specimen collected on an expired card or demographic information is missing or incorrect, then a repeat specimen must be requested as instructed per local laboratory processes. Unacceptable specimens adversely affect turnaround time and potentially delay the testing and referral of an affected infant.

Any screen positive result(s) should be acted upon as per relevant screen positive protocol(s) even if the result was obtained from an insufficient sample and / or where the result(s) cannot be confirmed in duplicate due to poor quality or insufficient sample for analysis. In addition, those samples where contamination is suspected for one the disorders for which we undertake weekend reporting, but cannot be confirmed in a timely manner then a clinical referral must be made.

3.1 Recording details on LIMS if a sample is unacceptable

Samples deemed unacceptable and the reasons why, must be recorded on LIMS to assist in queries for outstanding results and for performance management.

4.0 Appendix

1. **Effect of varying haematocrit (Hct) on DBS appearance. DBS with a low Hct are more symmetrical with smooth edges, in contrast those with higher Hct levels are smaller, darker and have an uneven edge.**

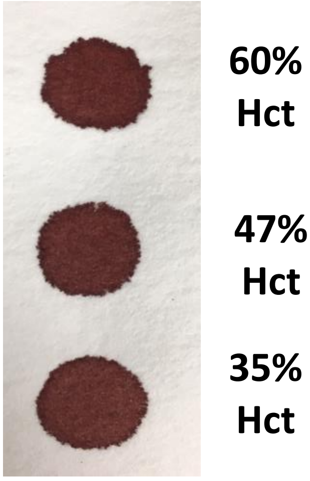

2. **Effect of contamination on producing falsely elevated screening analyte results.**

Analyte	Contaminant		
	Faeces	Infant feeds	Urine
IRT	↑↑↑		
Methionine		↑↑↑	
Phenylalanine		↑↑↑	
Tyrosine		↑	
Leucine/Isoleucine		↑↑↑	
C5-DC	↑↑	↑↑↑	↑↑↑
C5		↑	↑
C8	↑↑	↑	↑↑
C10	↑↑		↑↑

Table adapted from Winter T *et al*, Clin Chem Lab Med 2018.

Supplementary Figure 1.

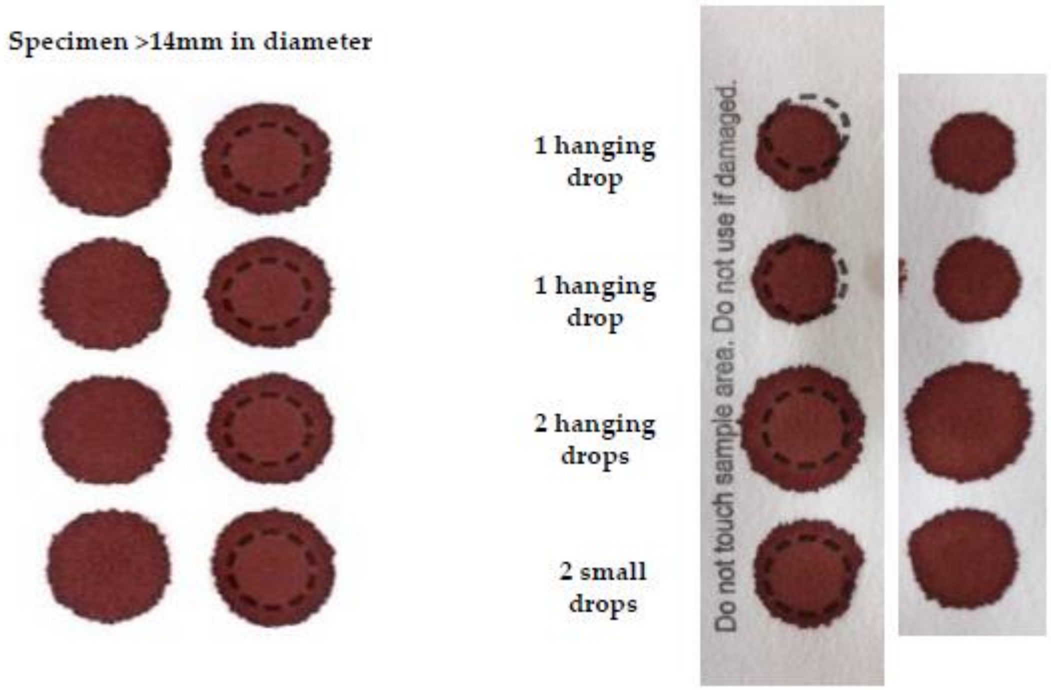

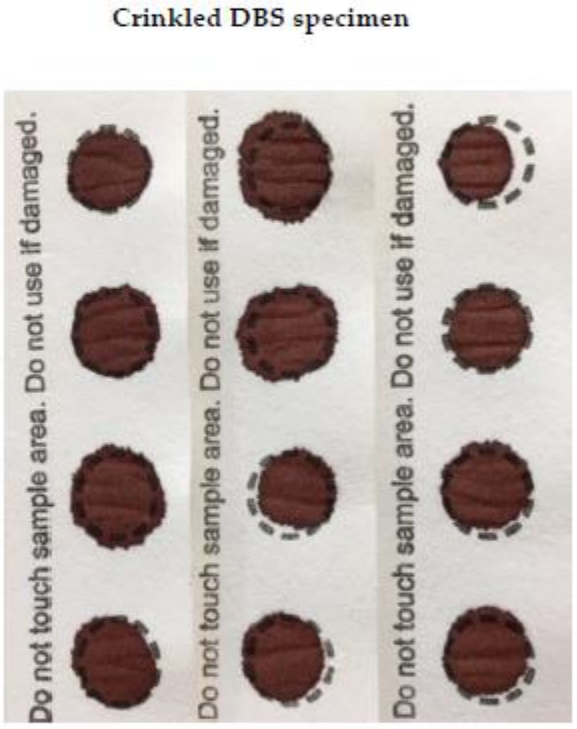

Poor application of blood leading to insufficient blood to fully saturate filter paper but large enough to allow two 3.2mm sub-punches to be taken.

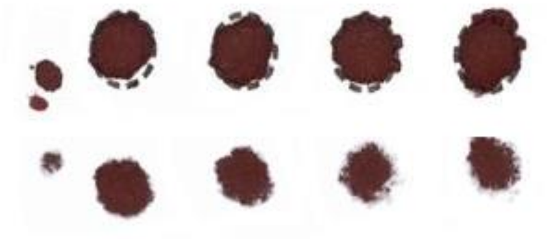

Multi-spotting of blood to fill the circle on the filter paper.

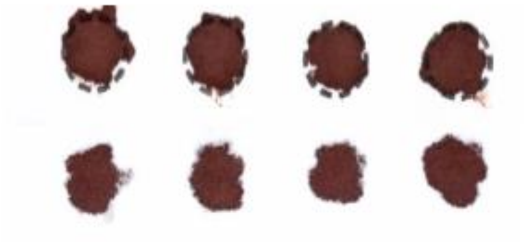

Supplement: Supplementary file 1 [file IJNS-10-00060-s001.zip › IJNS-3146113-supplementary.pdf]
